# Response measures influence comparative effectiveness of biological drugs in rheumatoid arthritis

**DOI:** 10.1186/1479-5876-10-S3-P52

**Published:** 2012-11-28

**Authors:** Helena Canhao, Vasco Romao, Fernando Martins, Joao Eurico Fonseca

**Affiliations:** 1 Rheumatology Research Unit, Instituto Medicina Molecular, Lisbon, Portugal

## Background

Rheumatoid arthritis (RA) is a chronic, disabling inflammatory disease. Tumor necrosis factor (TNF) and interleukin-6 (IL-6) are key elements in the disease pathway and constitute the target of the most commonly used biological drugs. Infliximab, adalimumab, etanercept and golimumab are TNF inhibitors and tocilizumab is an IL-6-receptor antagonist.

The aim of this work was to assess comparative effectiveness of biological drugs for RA patients registered in Reuma.pt, using different validate tools.

## Methods

We included RA first biological therapy users. Our primary outcome was EULAR good response at 6 months. Secondary outcomes were the proportion of patients in remission applying DAS28, CDAI and SDAI. Groups were compared using a multivariate logistic model.

## Results

520 RA patients. 123 treated with adalimumab, 204 etanercept, 31 golimumab, 110 infliximab and 52 with tocilizumab. Mean age at biologics start was 54.4±12.5 years and disease duration of 11.2±9.6 years. 86.9% were females and 83.0% were either rheumatoid factor or ACPA seropositive. Baseline DAS was 5.3±1.2 for adalimumab, 5.6±1.2 for etanercept, 5.4±1.3 for golimumab, 5.6±1.3 for infliximab and 5.7±1.2 for tocilizumab groups (p=0.12 ANOVA). At 6 months, DAS was 3.7±1.3 for adalimumab, 3.8±1.3 for etanercept, 3.5±1.3 for golimumab, 3.7±1.4 for infliximab and 1.6±0.99 for tocilizumab groups (p<0.0001 ANOVA). The probability of achieving EULAR good response at 6 months was modeled. Significantly higher rate of good response at 6 months was observed in tocilizumab compared to other groups with the exception of golimumab. DAS28 remission threshold was attained by a significantly higher number of patients treated with tocilizumab. Remission rates assessed by CDAI and SDAI did not show significant differences between groups.

## Conclusions

Tocilizumab demonstrated the best efficacy, but the difference varied with the disease activity tool chosen. These results reflect the importance of the tool used in the assessment of treatment response and may be explained by the pronounced effect of tocilizumab on the reduction of erythrocyte sedimentation rate, considered only by DAS28 and not by the other scores.

